# Situational Analysis of Cutaneous Leishmaniasis in an Endemic Focus of the Disease, Southeastern Iran

**Published:** 2018-03-18

**Authors:** Sajjad Fekri, Ahmad Ali Hanafi-Bojd, Yousef Salari, Parivash Davoodian, Reza Safari, Habib Dadvand, Mohsen Mohebbi, Hossein Issazadeh, Zahra Kamali

**Affiliations:** 1 Infectious and Tropical Diseases Research Center, Hormozgan University of Medical Sciences, Bandar Abbas, Iran; 2 Department of Medical Entomology and Vector Control, School of Public Health, Tehran University of Medical Sciences, Tehran, Iran; 3 Department of Diseases Control, Hormozgan University of Medical Sciences, Bandar Abbas, Iran; 4 Department of Diseases Control, Hormozgan University of Medical Sciences, Qeshm, Iran; 5 Department of Diseases Control, Hormozgan University of Medical Sciences, Jask, Iran

**Keywords:** Cutaneous leishmaniasis, Epidemiology, Spatial analysis, Iran

## Abstract

**Background:**

Leishmaniasis is one of the most important vector-borne diseases in Iran, existing in a variety of forms ranging from cutaneous to visceral forms. Jask County has been recognized as an endemic focus of the disease in the southeastern region of Iran. This study analyzed the situation of cutaneous leishmaniasis (CL) during 2006–2014.

**Methods:**

This cross-sectional analytical study was conducted on CL data got from health sector. ArcGIS 10.3 was exploited for the spatial analysis of CL. Potential high-risk areas of the disease regarding its' current geographical distribution were considered.

**Results:**

Overall, 874 CL cases had been registered in the district health center, implying an average incidence of 162.5per 100000. More than 90% of the cases emerged from rural areas. The disease is geographically distributed in the southeastern regions of Jask County. Over one-third of the total study area can be classified as high-risk areas, involving 61 villages with a total population of about 18000. Remarkably, altitude and total precipitation were realized to play key roles in CL transmission within the study area.

**Conclusion:**

Although the national protocol for the control of ZCL recommends the substantial destruction of rodent colonies serving as reservoirs of the disease in infected foci, critical improvement of the knowledge of the residents in these areas is crucial for community-based management of the disease in Jask County.

## Introduction

Leishmaniasis is a neglected disease which exists in three forms worldwide, cutaneous, visceral and mucocutaneous. It is among the 10 most important parasitic diseases in the tropics, and several dimensions of research supported by WHO have been conducted on various aspects of the disease (1). The disease is widely distributed in 90 countries, and its DALY is estimated as 2400000yr (2). Nearly 90% of cutaneous leishmaniasis in the world is originating from Afghanistan, Algeria, Brazil, Iran, Peru, Saudi Arabia and Syria, and the highest incidence of the disease occurs in Afghanistan and Iran (3). In Iran, the disease is commonly manifested in 17 out of the 31 provinces of the country, with estimated annual cases exceeding 20000 within the country (4). There has been an increasing trend of the prevalence of leishmaniasis in Iran, and provinces including Yazd, Bushehr, Khorassan-e-Razavi, Fars, Ilam, Khuzestan, and Esfahan have recorded the highest prevalence of the disease with many endemic foci (5).

The spatial distribution of CL depends highly on environmental factors, not excluding socioeconomic circumstances such as poverty and the knowledge of the entire populace concerning the transmission and prevention of the disease (6). Extensive drought occurrence within the last two decades, mostly in the eastern, central and southern parts of Iran, coupled with the development of urbanization and the extension of localized agricultural activities in some other areas, have provided suitable conditions for the transmission of the disease because of the proximity between human, reservoir hosts, and vectors. *Phlebotomus papatasi* is identified as the main vector of zoonotic cutaneous leishmaniasis (ZCL), although *Ph. salehi* has been infected with *L. major*, the causative agent of ZCL, in some areas (7, 8).

Gerbils (Rodentia: Gerbillidae) are the main reservoir hosts of the diseases in Iran (9). Earlier, in the Jask County, southeastern Iran, *P. papatasi* and *Meriones hurrianae* were reported as the vector and the reservoir of CL respectively, and have detected *L. major* infection in *Gerbillus nanus* (10). Recently, this county has been targeted as the chief endemic focus of zoonotic cutaneous leishmaniasis (ZCL) in the Hormozgan province of Iran.

Although no mortality associated with CL has been recorded so far, the disease is heavily problematic for the inhabitants living in endemic areas due to the debilitating and disfiguring effect of the disease, and the long latency period once the disease is established. Regardless of the pervasive efforts to prevent the disease, no potent vaccines have been developed for the disease, leaving its treatment complicated (11). The best alternatives for controlling the disease are environmental management adoption, personal protection and the control of rodents (reservoir) of the disease (12). As a zoonotic vector-borne disease, leishmaniasis is greatly affected by environmental and socioeconomic factors which play central roles in the disease cycle. Using geographical information system (GIS) these factors can be investigated to detect the high-risk areas of CL infection, and to rank effective measures in controlling the disease (13, 14).

Recently, GIS has been applied in similar studies to model the probability of presence for the vectors (8) and reservoirs (9) of ZCL, and to produce a risk map for the disease using gathered data of the disease and associated environmental factors (14–16). GIS-based techniques are potentially beneficial to manage ZCL in different settings.

Considering the current significant situation of cutaneous leishmaniasis in the Jask County, the present study was aimed at assessing the epidemiology of the disease and analyzing its potential risk of infection.

## Materials and Methods

**Fig. 1.**
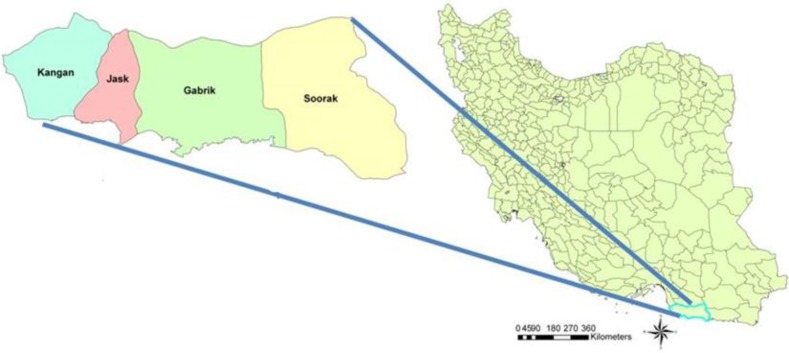
Jask County in southeast of Iran

Jask County, encompassing a landmass of 11141km ^2^ , is located in the eastern quarter of the Hormozgan Province, southeastern Iran (Fig. 1). The geographical coordinates of the county lie between 57°10′–59 ° 16′ E and 25°23′–26°13′ N. As at 2015, the population of Jask County had reached 53770. The county comprises 164 residential villages. The weather of this county is described as being warm and dry in warmer summers and cold in temperate winters although the relative humidity across the coastal plain exceeds 50%. Jask County is often designated as having a hot desert climate. Topographically, this county has two distinct areas; a coastal plain mainly covered by the city of Jask and most villages bordered with the Oman Sea to the south, and a hilly to mountainous area in northern part.

### Data collection

Characteristic features of the cases of cutaneous leishmaniosis referred to the appropriate health division for treatment were documented in special forms designed by the ministry of health. In this descriptive-analytical study, necessary data were passively extracted from the above-mentioned forms during 2006–2014. Data were later transferred to an excel spreadsheet and the related charts and tables were computed using this software.

### Ethical consideration

This study has been ethically approved by the Infectious and Tropical Diseases Research Center of the Hormozgan University of Medical Sciences in Bandar Abbas, Iran. The confidentiality of the records of patients was assured.

### Spatial analysis

Data on the cases of cutaneous leishmaniasis were identified in specific inhabited places of the villages displayed on the base maps provided by the National Cartography Organization. Shapefiles of the distribution of CL-positive cases were prepared and classified using ArcGIS 10.3. Raster layers of annual average of mean daily temperature (°C) and relative humidity were prepared by Inverse Distance Weighted (IDW) analysis at spatial resolution of 50m ^2^ /pixel, using the data provided by different synoptic stations, including Jask and 8 other nearby cities. These data were collected by the National Meteorological Organization for research in the country.

Kriging and co-kriging are geostatistical techniques used for interpolation (mapping and contouring) purposes. Both methods are generalized forms of univariate and multivariate linear regression models, for estimation at a point, over an area, or within a volume. They are linear-weighted averaging methods, similar to other interpolation methods, however, their weights depend not only on distance but also on the direction and orientation of the neighboring data to the unsampled location. Co-kriging can be seen as a point interpolation, which requires a point map as input and which returns a raster map with estimations and optionally an error map. Co-kriging is a multivariate variant of the ordinary kriging operation: Co-kriging calculates estimates or predictions for a poorly sampled variable (that we want to predict; in this study: CL cases) with help of a well-sampled variable (the covariable). The variables should be highly correlated (positive or negative). Therefore, we used CL cases in the study area as sampled variable (17). Some variables such as mean temperature, total precipitation, altitude and Normalized Difference Vegetation Index (NDVI) were highly correlated with CL incidence. Therefore, we used the first three independent variables for prediction the risk of CL as dependent variable. The prediction map was classified using natural breaks into five classes for infection chance.

## Results

A total of 874 CL cases were discovered by the health center of Jask district during the study period. The highly infected age group belonged to 10–19yr, with a frequency of 21.4% (Table 1). The majority of cases which reported from the 87 residential places came from the rural areas (91%). Analysis of the lesions on the different parts of the body showed 62.3% of the lesions occurred on the hand/foot, 28.6% were presented on the face, and 9.1% on the remaining parts of the body. The number of lesions present was also counter-checked, and most of the patients (56%) suffered one lesion each on their body, followed by two (22.4%), and more than 2 lesions (21.6%). Residents of rural areas of the county were more infected than those of urban areas, such that 91% of cases arose from the villages (Table 1).

**Table 1.** Some demographic information of patients infected by cutaneous leishmaniasis in Jask County, Southeastern Iran, 2006–2014

**Year**	**Gender**		**Age groups (yr)**							**Residence place**	

	**Female**	**Male**	**0–4**	**5–9**	**10–19**	**20–29**	**30–39**	**40–49**	**≤50**	**Urban**	**Rural**
**2006**	106	115	49	36	56	30	12	14	11	14	207
**2007**	119	117	50	46	51	36	21	12	20	27	209
**2008**	83	112	36	43	49	27	13	9	19	14	182
**2009**	41	64	26	24	19	16	9	7	4	11	94
**2010**	13	26	7	10	6	11	2	2	1	2	37
**2011**	10	15	5	9	4	4	3	0	0	6	19
**2012**	0	7	1	0	3	1	0	1	1	0	7
**2013**	15	15	12	5	7	2	3	1	0	0	30
**2014**	10	6	1	6	5	0	1	1	2	4	12
**Total**	397	477	187	179	200	127	64	47	58	78	797

**Fig. 2.**
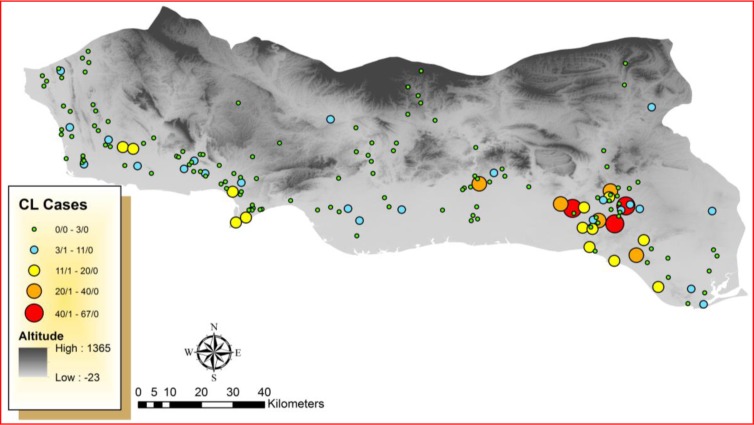
Spatial distribution of cutaneous leishmaniasis in Jask County, Southeast of Iran

Spatial distribution of CL cases across the area showed the highest morbidity in the southeastern regions (Fig. 2). Overall, the disease was recorded in 87 localities, including villages and some quarters of the urban centers.

**Fig. 3.**
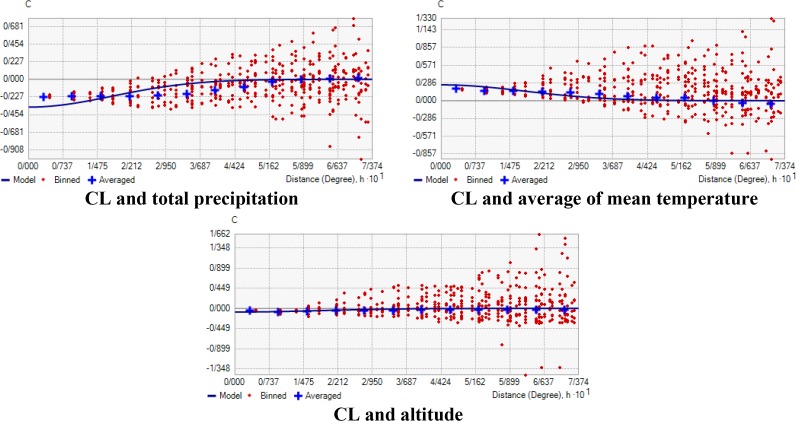
Experimental anisotropic semivariograms for cutaneous leishmaniasis X-axis is distance times 10 ^1^ (in Degree), Y-axis is semivariance for CL cases located between specified distances

Cokriging analysis involving altitude, mean monthly temperature and total annual precipitation variables showed a positive autocorrelation between the disease and the total precipitation and altitude, as presented in the experimental semivariograms (Fig. 3). The distance where the model first flattens out is known as the range. Locations separated by distances closer than the range in Fig. 3 are spatially autocorrelated, whereas locations farther apart than range are not.

**Fig. 4.**
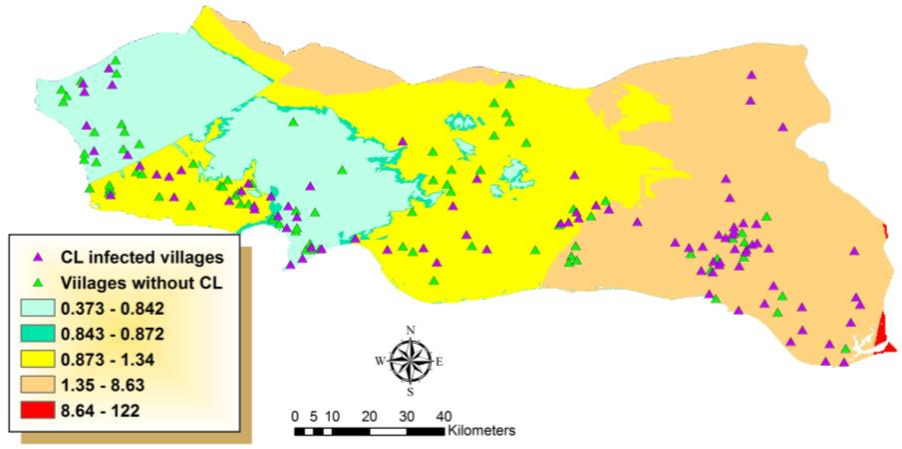
Cokriging prediction for the risk of infection to cutaneous leishmaniasis in Jask County, Southeast of Iran

**Fig. 5.**
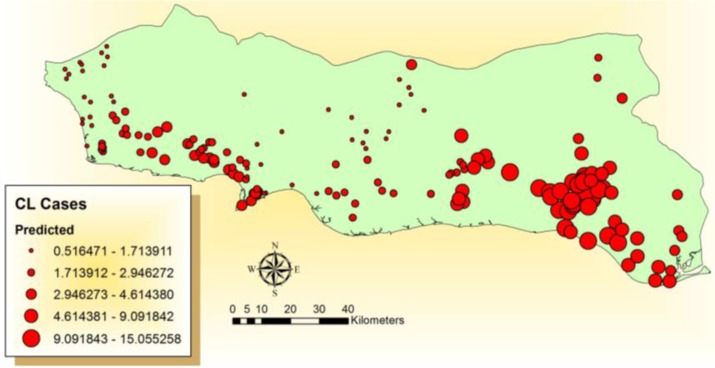
Cokriging prediction for the risk of infection to cutaneous leishmaniasis in different villages of Jask County, Southeast of Iran

The prediction map indicated the eastern parts of Jask County, together with some northern areas of the county, are more susceptible to maintaining CL transmission (Fig. 4). Totally, 61 rural/urban areas were categorized as high-risk areas for CL transmission, with a total population of closely 18000. Conversely, more than a third of Jask County inhabitants are living in high-risk areas, while the risk of CL transmission is medium in more than 50 rural/urban areas. According to the model prediction for ZCL in villages of the study area, the highest probability of the disease has predicted for southeastern area, followed by southwestern and central parts (Fig. 5).

## Discussion

Jask County, in the southeastern region of Iran, is an endemic focus of both malaria and leishmaniasis (18–21). Although in recent years few indigenous cases of malaria have been reported from this area, cutaneous leishmaniasis is the main vector-borne infectious disease with an estimated incidence of 162.5per 100000 population. From 2006 to 2009, the disease erupted into an epidemic condition in some areas of the county, however, this situation was brought under control which led to a decreasing trend in the incidence of the disease after the year 2009. Correspondingly, this decreasing trend was consistently observed nationwide regarding CL infection in Iran (5). A major determinant contributing to this trend is drought, because of climate change (22), a problem experienced by the country for several years. Children were the most infected group in this county, as seen in other endemic foci of the disease throughout the country (5), and the commonly occurring cases were noticed in those belonging to 0–4 and 5–9yr age groups.

The zoonotic form of cutaneous leishmaniasis has been known to be circulating in the Jask County, and *L. major* has been detected in the sand fly vector (*Ph. papatasi*) and gerbils (*Tatera indica* and *Gerbilus nanus*), as well as human lesions (18, 19). This form of the disease has been reported in Baluchistan area, bordered to the east of our study area. Verily, ZCL transmission is extended from Baluchistan to Jask, following an epidemic which occurred in the 2000s in some counties of Baluchistan (from Dashtyari to Chabahar, and Konarak) (23). Another great concern in the epidemic of ZCL in Jask is that may occur because reducing vector control operations including indoor residual spraying (IRS) in the active foci following a sharp drop in malaria cases (11).

Water transfer project at Jagin Dam, in the north of Jask County, has been recently considered to develop agriculture in this county. Considering the living habitat of *T. indica*, the main reservoir of ZCL in the study nesting in barren lands around the crop fields, and the presence of suitable ecological niches for this species and other vectors in southern Iran (8, 9) a new upsurge of the disease should be expected because the lack of adequate attention to preparedness and mitigation. Our prediction for the risk of CL showed one-third of the land-mass of the county has a higher potential for CL transmission. This is clear in our results whereby a population of about 18000 is living in 61 villages/urban areas which are at risk of ZCL infection.

Cokriging is an accurate method for spatial interpolation. Considering the co-variates that affect the transmission of a disease, targeting variables of the weather proven to correlate well with the disease transmission would be cost-effective, rather than focusing on field data collection and sampling. Previously, the transmission of ZCL was found to be dependent ona number of climatic factors including higher temperature, lower relative humidity, lower total rainfall, higher evaporation and lower number of rainy days (14, 24). Thus, this analysis was used to cross-correlate environmental variables with the records of CL in predicting high-risk areas. Experimental anisotropic semivariograms in our study showed increasing trend between the disease occurrence with total precipitation, negative trend between CL occurrence and mean temperature, while for altitude it had no increase or decrease. Because the distances between sampling points had no significant differences regarding three environmental variables used in this study, there was no difference between sampling points (Fig. 3). By the way, this model predicted eastern regions of the Jask County are more vulnerable to establishing new endemic foci. Likewise, this method has been used to estimate the habitat suitability for *Ixodes scapularis*, the vector of Lyme disease (25), *Boophilus microplus*, a serious cattle pest (26), and dengue vector population (27).

Hence, this technique will be essential for later studies on leishmaniasis. According to the spatial correlation between three environmental variables used in this study, it is predicted cases of ZCL will increase in some villages of Jask County (Fig. 5). Besides, to prevent the disease expansion to the new areas, it is necessary to do some preventive measures such as community-based health education programs, environmental sanitation and personal protection against sand fly vectors.

## Conclusion

Cutaneous leishmaniasis is an endemic disease in Jask County and should be considered by health sector authorities, particularly in high-risk areas explored in this study. Massive development projects begun in this area will provide job and trading activities for people emigrating from non-endemic areas of the, and from some other countries. This situation is likely to increase the morbidity associated with the disease due to the invasion of non-immunized immigrants. Therefore, preventive measures along with health education and promotion will help curtail the risk of infection and morbidity.
